# Two Strategies for Researching the Endangered Yangtze Finless Porpoises Suggest Data‐Poor Areas Are Worthy of Greater Conservation Efforts

**DOI:** 10.1002/ece3.71649

**Published:** 2025-06-26

**Authors:** Yi Lu, Hongyan Bi, Weijie Jiang, Chong Cui, Jing Zhu, Pei Wang, Xinrong Xu, Guang Yang, Wei Chen, Zhengfei Wang

**Affiliations:** ^1^ Southern Marine Science and Engineering Guangdong Laboratory (Guangzhou) Guangzhou Guangdong China; ^2^ School of Wetlands Yancheng Teachers University Yancheng Jiangsu China; ^3^ College of Life Sciences Henan Normal University Xinxiang Henan China; ^4^ College of Bioscience and Biotechnology Yangzhou University Yangzhou Jiangsu China; ^5^ Jingjiang Ecological Environment Bureau of Taizhou City Taizhou Jiangsu China; ^6^ Jiangsu Key Laboratory for Biodiversity and Biotechnology, College of Life Sciences Nanjing Normal University Nanjing China

**Keywords:** abundance, conservation, distribution, Yangtze finless porpoises

## Abstract

The distribution and abundance of species are crucial components of their population ecology and serve as the foundation for effective conservation efforts. However, baseline data may be insufficient in areas where surveys have been inadequately conducted, complicating the implementation of protective measures. In this study, we employed two complementary approaches to gather baseline information on the endangered Yangtze finless porpoise in the Jingjiang section of the Yangtze River. Firstly, we gathered information about porpoises from a questionnaire survey (fishers). The results indicate that porpoises have been encountered over the past few decades, suggesting that this area has always been the habitat of porpoises. Secondly, boat based visual line transect surveys were carried out from September 2021 to August 2024. These findings validated local fishers' ecological knowledge by demonstrating the presence of a substantive population inhabiting Jingjiang section. The abundance was estimated to approximately 39 individuals, which exceeds previous estimates. Additionally, we identify the core distribution area of finless porpoises in the Mucheng Park waters. Overall, this study has closed knowledge gap about porpoises in the region. The results provide a scientific foundation for the management and conservation of porpoises.

## Graphical abstract

Two strategies were used to assess the abundance and distribution of Yangtze finless porpoises in the Jingjiang section of the Yangtze River to make up for the lack of knowledge about this area. These results provide support for the management and conservation of finless porpoises.

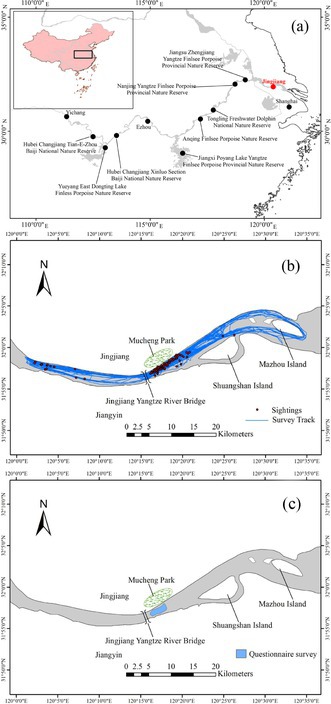

## 1 Introduction

When developing conservation strategies for endangered species, timely and accurate monitoring of species distribution patterns and population dynamics is fundamental (Evans and Hammond 2010). In addition, surveys and filling research gaps are crucial for declining populations (Wilson and Delahay 2001). For example, research on baiji started late, with many knowledge gaps and limited funding and technical support, resulting in insufficient understanding of its habits and population dynamics, which hindered effective conservation efforts (Turvey et al. 2007). Specifically, the Baiji population declined rapidly during the 1980s and 1990s, and by 1981 was estimated at 400 individuals (Turvey et al. 2007). However, little effort was ever made to implement the baiji recovery programme. The species consequently declined to just 13 individuals by 1999 (Zhang et al. 2003), and was declared functionally extinct in 2006 (Turvey et al. 2007). Therefore, it is both urgent and necessary to address research gaps in the distribution and dynamics of endangered species for conservation.

The Yangtze finless porpoise ( *Neophocaena asiaeorientalis* ), a critically endangered species endemic to China, is exclusively distributed in the middle and lower Yangtze River mainstream and its two largest connecting lake (Poyang Lake and Dongting Lake). Over the past years, overfishing, vessel traffic, and pollution in the Yangtze River Basin have led to a decline in porpoise populations, leading to its classification as critically endangered on the IUCN Red List. Surveys conducted between 1984 and 1991 estimated the Yangtze finless porpoise population at around 2700 individuals, with 2550 in the main channel of the Yangtze River, 104 in Dongting Lake, and 52 in Poyang Lake, indicating a continuous distribution from Yichang to the Yangtze estuary (Zhang et al. 1993). Between 1997 and 1999, the population decreased to approximately 2000 individuals (Mei et al. 2011). In the 21st century, systematic surveys employing the line transect method have continued, initiated by Xiao and Zhang (2000). By 2006, the population of finless porpoise was estimated at around 1800 individuals, indicating a persistent decline (Zhao et al. 2008). Only 1045 individuals remained in 2012, showing a significant decrease over the years (Mei et al. 2014). The decline had slowed until 2017, with 1012 individuals (Huang et al. 2020). This trend suggested that conservation efforts may be taking effect. Recognizing the urgent need for further protection, the Yangtze finless porpoise was upgraded to a first‐class nationally protected wild animal in 2021 (Mei et al. 2021). In 2022, a survey estimated the population to approximately 1249 individuals, with 595 in the main channel, 492 in Poyang Lake, and 162 in Dongting Lake, representing an overall increase of 23.42% compared to 2017, with an annual growth rate of 4.30%.

**FIGURE 1:**
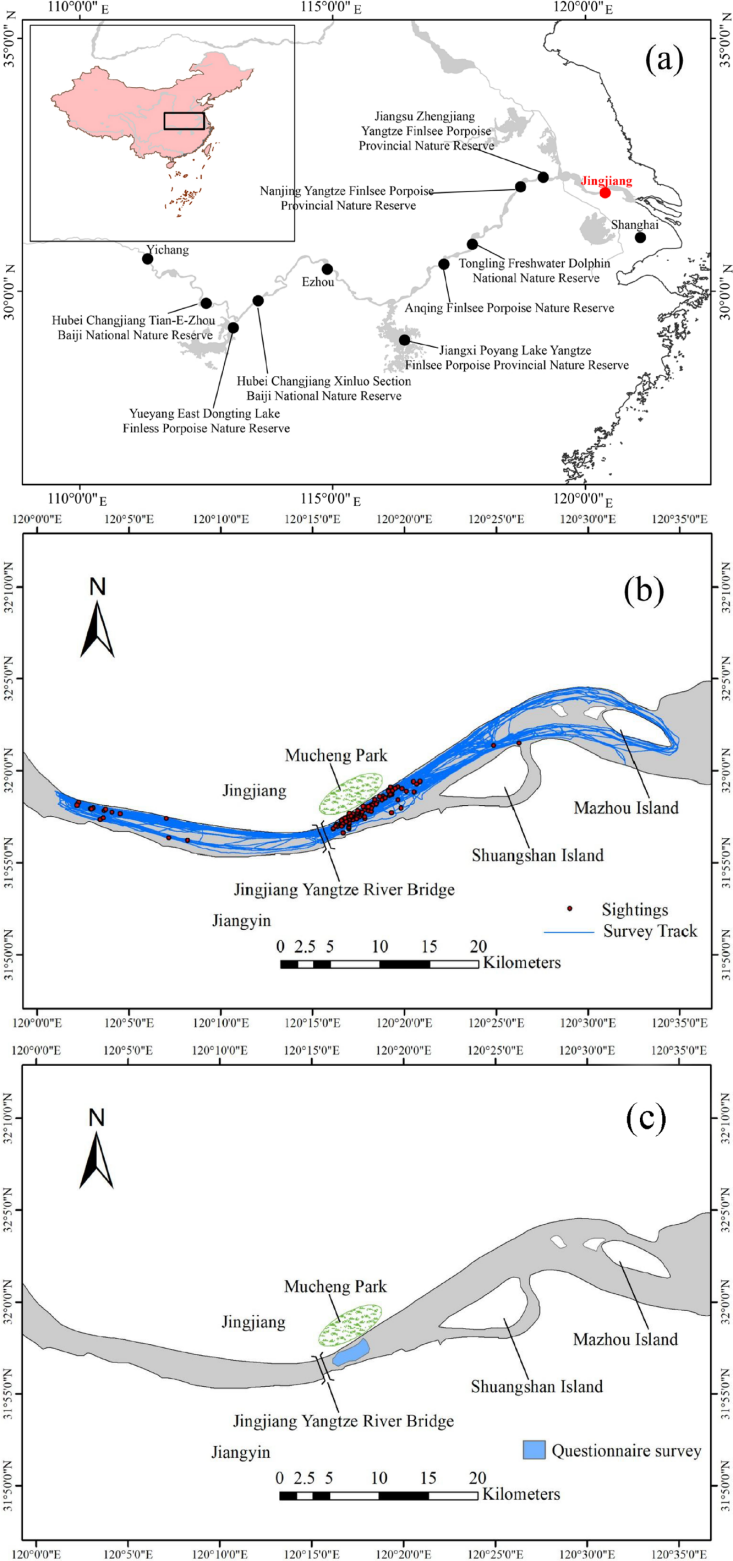
(a) Distribution range of Yangtze finless porpoises and locations of nature reserves; (b) Map of Jingjiang section of the Yangtze River, displaying survey tracks and Yangtze finless porpoises group sightings between September 2021 and August 2024; (c) The distribution of Yangtze finless porpoises reported by previously local fishers.

Since the 1980s, three major conservation measures have been proposed for the Yangtze finless porpoise in China: in situ conservation, *ex‐situ* conservation, and artificial breeding. Currently, eight in situ reserves have been established by the Chinese government (Figure 1a), namely Honghu (or Xinluo) in Hubei (1987), Shishou (or Tian‐e‐Zhou) in Hubei (1990), East Dongting Lake in Hunan (1996), Tongling in Anhui (2000), Zhenjiang in Jiangsu (2003), Poyang Lake in Jiangxi (2004), Anqing in Anhui (2007), and Nanjing in Jiangsu (2014) (Lu et al. 2023), covering 40% of key habitats and 80% of the population (Shan 2019). Besides, *ex‐situ* reserves also play important roles in conservation, with *ex‐situ* populations now exceeding 150 individuals. The artificial breeding of the Yangtze finless porpoise is an effective way for preventing the species extinction. A significant milestone was achieved in 2005 with the birth of the first captive‐born Yangtze finless porpoise, named “Tao‐Tao” (Wang et al. 2005). Subsequently, over a dozen individuals have been successfully born (Hao et al. 2023), and in 2017, captive‐bred individuals were successfully reintroduced into the wild (Song et al. 2017).

The Jingjiang section, located in the lower reaches of the Yangtze River, in Jiangsu Province, China, has long been recognized as a low‐density area for finless porpoises (Huang et al. 2020), and serves as a vital ecological corridor in the lower reaches of the Yangtze River (Figure 1a). Protecting this region is essential for maintaining the integrity of the lower Yangtze porpoise habitat. Studies and monitoring of the finless porpoise population and habitat in this area are critical for species conservation and recovery. Recent research indicates that human activities continue to threaten the survival and habitat quality of the finless porpoise in Jiangsu Province. These threats include underwater noise from shipping (Li et al. 2024) and the operation of cascade dams in the upper Yangtze River (Wang et al. 2022). Additionally, the finless porpoise has long been threatened by climate change in the Yangtze River Basin (Hao et al. 2024). For example, the freezing of the water surface of Tian‐e‐Zhou Oxbow in 2008 and the drought of Poyang Lake in 2012 both resulted in mortality events of the Yangtze finless porpoise (Hao et al. 2024). In addition, rescue operations were implemented for Yangtze finless porpoises in Tian‐e‐Zhou Oxbow and Poyang Lake during the 2022 extreme drought event (Hao et al. 2024). Consequently, research on the population and habitat of the finless porpoise in this section is both necessary and urgent.

In this study, we conducted questionnaire and visual line‐transect surveys in the Jingjiang section of the Yangtze River from September 2021 to August 2024. The survey documented finless porpoise occurrence locations and distribution. The results close knowledge gaps on finless porpoises in the Jingjiang section, providing current population estimates and offering recommendations for further conservation and research.

## 2 Methods

### 2.1 Study Area

The river width in the Jingjiang section ranges from 1.3 to 6 km. The narrowest and widest sections are located at the Jiangyin Bridge and Shuangshan Island, respectively. The survey covered approximately 52 km in length. Within the survey area, there are two sandbanks, Mazhou Island and Shuangshan Island. The water depth of the main channel is 10 to 15 m, and the maximum depth can reach 25 m. Water levels are higher in the spring and summer and lower in the autumn and winter. This section contains multiple ports and ferry terminals with intensive ship traffic, including freighters, pleasure boats, ferries, high‐speed vessels, and other engineering vessels.

### 2.2 Questionnaire Survey

The questionnaire survey was conducted anonymously in September 2021, with 45 respondents contacted through local fisheries management authorities (these fishers had ceased Yangtze River fishing operations due to the 2021 fishing ban policy). We extracted information on the fishery (locations and times) and all porpoise‐related data from the questionnaire survey, namely number of sightings, times, distance from shore, number of individuals sighted, group size, and frequency of calf (estimated based on body size) sightings.

### 2.3 Line Transect Method

From September 2021 to August 2024, boat‐based surveys were conducted in the Jingjiang section of the Yangtze River (Figure 1b). Based on previous studies using water level as the classification standard, the year is divided into four seasons: spring (March–May), summer (June–August), autumn (September–November), and winter (December–February) (Lu et al. 2023). Line transect surveys were performed using a survey boat measuring 17.5 m in length and 4 m in width with a 1 m observation platform. The boat traveled parallel to riverbanks during clear weather with calm waters (Beaufort scale ≤ 3). The survey team consisted of at least four members: three on the foredeck (primary observation team, a left observer, a recorder, and a right observer) and one (conditionally independent observer) on the aft deck of the boat. All observers were trained beforehand and three observers had previously participated in other cetacean surveys. The left and right observers used 7 × 50 mm Fujinon binoculars, supplemented by naked‐eye observations, to cover a 100° field of view on their respective sides, from 90° to slightly past the centerline by 10°, ensuring overlap for detecting porpoises at the centerline. Observers used binoculars for over 90% of the survey time, while the recorder was responsible for recording data and observing the 90° arc on either side using the naked eye. This arrangement ensured that porpoises near the survey vessel were also detected. The independent observer did not communicate with primary observers and only recorded porpoises missed by the primary observer. Team members rotated roles every 30 min to maintain high levels of attention throughout the survey (Zhou et al. 2016).

The survey vessel followed predetermined routes at approximately 10 km/h (approximately 5 knots). When a finless porpoise was sighted, all team members participated in observation and data collection, with details promptly communicated to the recorder. In suitable areas, a drone was used to observe porpoise behavior from an aerial perspective. Information was collected on: time and location of sightings, distance from the vessel, angle relative to the survey line, group size and structure, distance from shore, and surrounding environmental conditions. We calculated the perpendicular distance by recording the radial distance and bearing angle (Zhao et al. 2008). The radial distance between the porpoise and the boat was estimated by referring to buoys and other floating objects around the porpoise, and the bearing angle was measured using a digital protractor. The farthest 5% of all data were truncated to assess population abundance (Focardi et al. 2002). To minimize observer bias in abundance estimation, three observers conducted simultaneous but separate group size assessments (Barlow 1995; Barlow and Forney 2007). One observer performed auxiliary observations using a digital camera (Canon‐1D X Mark II), while the remaining two team members recorded the group size with the naked eye. After each observation, observers immediately cross‐validated their independent group size estimates. A consensus‐based approach was implemented whereby only concordant counts (defined as ≥ 2 observers reporting the same numerical value) for abundance evaluation. If the porpoise exhibited evasive behavior, tracking and photographing were stopped, and observations continued from a distance. If no evasive behavior was observed, tracking and photographing continued until the porpoise disappeared or all individuals in the group were clearly photographed, after which the survey vessel resumed the route.

### 2.4 Distribution and Habitat Range Estimation

Two indicators were calculated to measure the distribution of porpoises: group sighting density and distance from shore (Liu et al. 2020). The group sighting densities were the number of sightings of porpoises within a fixed range, weighted by the survey effort in each grid cell. To calculate the group sighting density, the survey area was divided into 0.005° × 0.005° grid cells. Kernel density estimation was used to analyze the spatial distribution patterns of porpoises. Furthermore, to assess the habitat range and core habitat of the Yangtze finless porpoise in the Jingjiang section, this study employed two commonly used methods: the Minimum Convex Polygon (MCP) method and the Kernel Density Estimation (KDE) method. The MCP is the smallest polygon in which no internal angle exceeds 180° and which contains all the sites of occurrence. The MCP range represents the minimum habitat range of the porpoises (Wang et al. 2017). The 95% KDE range was used to identify the majority of the locations used by the porpoises, and the 50% KDE range to identify the range of the core habitat (Sprogis et al. 2016). All analyses were performed in ArcMap10.2.

### 2.5 Abundance Estimate

To estimate the abundance of porpoises, the software Distance (version 7.1) was used with the Conventional Distance Sampling analysis method. The method employs a robust key function plus series expansion (adjustment term) to fit the data. Candidate key functions include Half‐normal, Hazard‐rate distributions, and Negative polynomial, while adjustments can be expressed as Cosine terms, Hermite polynomials, or Simple polynomials. The best model was selected based on the lowest value of the Akaike's Information Criterion (AIC). The goodness‐of‐fit for the best model was evaluated through Q‐Q plot and the weighted (to give higher weight to distances near zero) chi‐square test, following the methodologies of Burnham et al. (2004) and Stober and Smith (2010), with statistical significance (*p* < 0.05) indicating inadequate model fit. The porpoise abundance (*N*) was calculated using the following formula:

$$N=A\times \hat{D}=A\times \text{2L}\;g\left(0\right)\;n\;\hat{f}\left(0\right)\;S$$

where (A) is the area of the survey region (km^2^), ($\hat{D}$) is the estimated porpoise density in the survey area (porpoises/km^2^), (L) is the total length of the transect lines (km), (g(0)) is the probability of detecting a group on the transect line, (*n*) is the number of groups sighted, $\left(\hat{f}\left(0\right)\right)$ is the sighting probability density on the trackline, (S) is the estimated mean group size.

This model was fitted using maximum likelihood with ungrouped perpendicular distances. In addition, g(0) was evaluated using the method of Barlow (1995). The formula is as follows:

$$g\;\left(0\right)=1-\frac{n_{2\omega }f_{2}\left(0\right)}{n_{1\omega }f_{1}\left(0\right)}$$

where g(0) indicates the probability of detecting a group directly on a trackline by primary observation team; $n_{1\omega }$ and $n_{2\omega }$ indicate the total number of groups seen within the truncation distance by primary observation team and conditionally independent observer, respectively; $f_{1}\left(0\right)$ and $f_{2}\left(0\right)$ indicate the sighting probability density on the trackline by primary observation team and conditionally independent observer, respectively.

## 3 Results

### 3.1 Questionnaire Survey

During the questionnaire surveys, we interviewed 45 local residents living near the surveyed waters. Of the 45 respondents, eight reported porpoise observations. All of these respondents were fishermen who had worked in the Jingjiang section prior to the implementation of the fishing ban policy in 2021. They were aged between 45 and 60 years, with 20–30 years of fishing experience. The fishing vessels they used were approximately 20 m in length. According to their accounts, finless porpoises were often sighted, ranging from below the Jingjiang Yangtze River Bridge to the Mucheng Park, at a distance of approximately 200–800 m offshore (Figure 1c). Most sightings occurred between April and May, with occasional sightings in August and September. The group size was typically 5–6 individuals, with calves predominantly observed in April and May each year.

### 3.2 Boat Survey Summary

**FIGURE 2:**
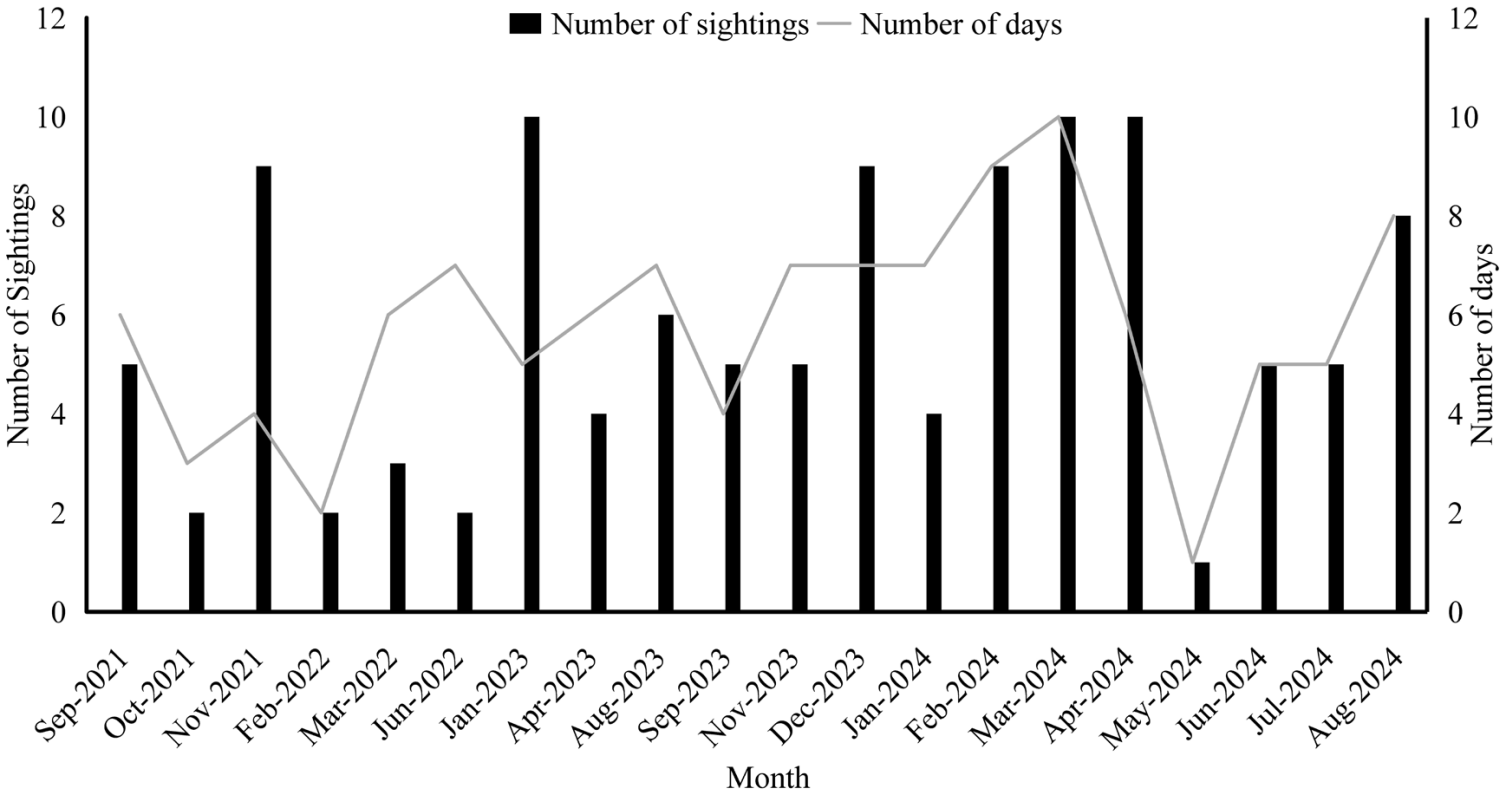
Number of survey days and number of sightings of Yangtze finless porpoises between September 2021 and August 2024.

During the four‐year survey period (September 2021–August 2024), a total of 115 survey days (Figure 2) were conducted, covering 4690 km (Table 1). Surveys were conducted during 29, 32, 24, and 30 days in the spring, summer, autumn, and winter, respectively (Figure 2). To enhance survey accuracy, the survey days encompassed the majority water areas of the Jingjiang section, ensuring comprehensive monitoring of the Yangtze finless porpoises habitat range. A total of 114 groups of Yangtze finless porpoises were sighted during the surveys (Figure 2), and the mean encounter rate was 0.024 groups/km (Table 1). Group sizes ranged from 1 to 16 individuals, with a mean group size of 3.91 ± 2.88 individuals.

**TABLE 1:** Effort, sightings and encounter rate of Yangtze finless porpoise groups in Jingjiang section between September 2021 and August 2024.

Year	Days (no.)	Survey track(km)	Sightings (no.)	Encounter rate (km^−1^)
2021	13	565	16	0.028
2022	15	684	7	0.010
2023	36	1360	39	0.029
2024	51	2082	52	0.025
Total	115	4690	114	0.024

### 3.3 Distribution and Habitat Range Estimation

**FIGURE 3:**
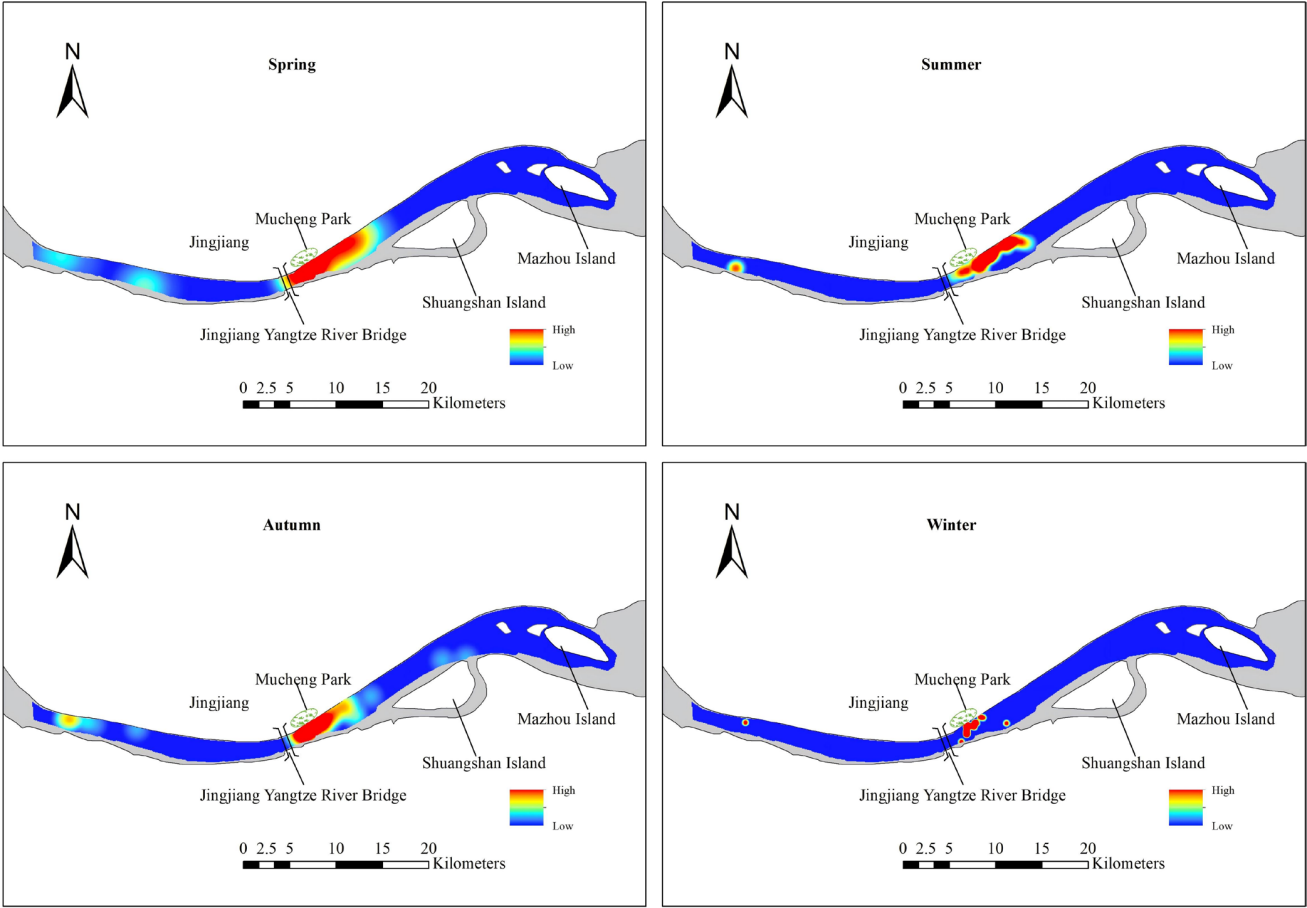
Distribution pattern of Yangtze finless porpoises in Jingjiang section. The heat map of distribution based on the sightings using kernel density analysis in ArcMap 10.2. Spring: March to May, Summer: June to August, Autumn: September to November, Winter: December to February.

**FIGURE 4:**
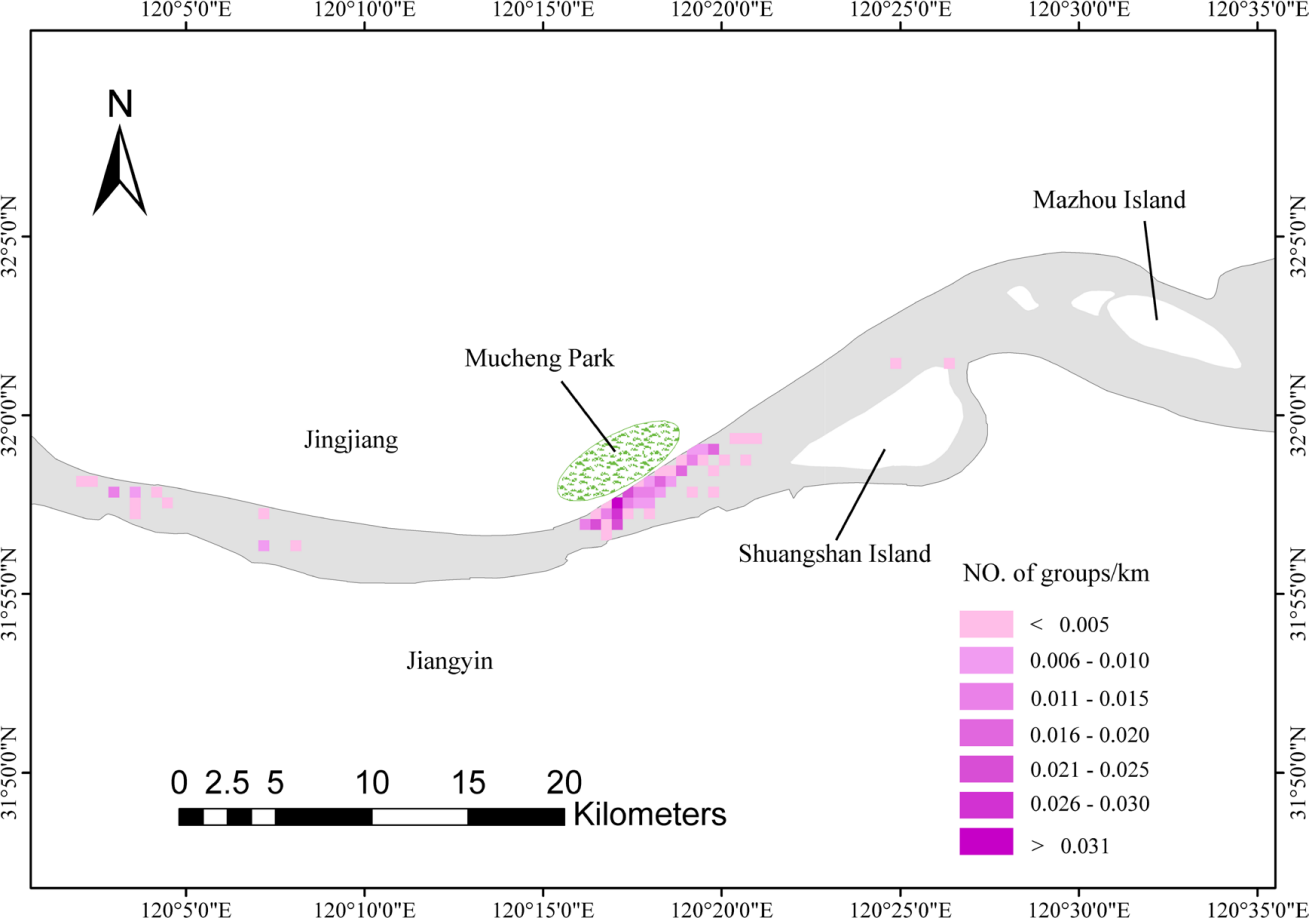
Group sighting density weighted by survey effort in each grid‐cell.

**FIGURE 5:**
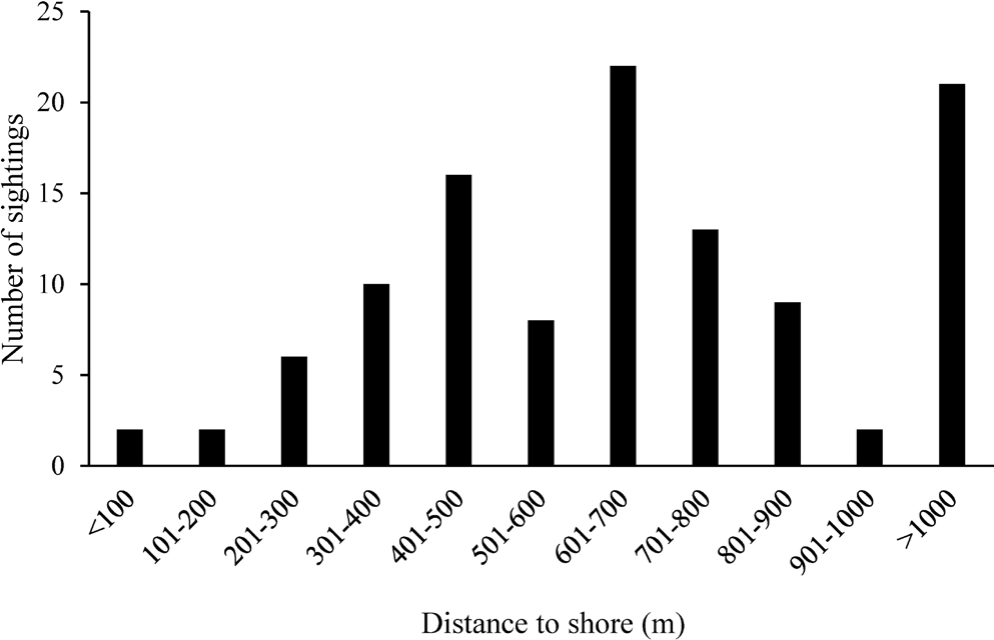
Frequency distribution of sightings of Yangtze finless porpoises off the riverbank in Jingjiang section between September 2021 and August 2024.

The high‐density range showed incomplete spatial consistency across seasons. However, in all seasons, porpoises were all mainly concentrated between Jingjiang Yangtze River Bridge and Shuangshan Island, particularly near Mucheng Park (Figure 3). These areas have less anthropogenic impacts. By evaluating the sighting sites, a high density of sightings was mainly observed in some concentrated grid cells near Mucheng Park (Figure 4). In addition, the porpoises were encountered within 2000 m from shore, in all 114 recorded distances to shore, and only two were less than 100 m (Figure 5). The highest proportion of records were observed within 600 to 700 m, with a total of 22 sightings (21.12%) (Figure 5). Relatively high sighting frequencies were also recorded at distances of 300–400 m and 700–800 m, with 10 (9.61%) and 13 (12.50%) sightings, respectively (Figure 5). There were 75 sightings within 200–800 m, accounting for 72.12%, which was consistent with the information provided by fishermen (Figure 5). These results indicated that the porpoises exhibit a habitat preference, generally favoring areas at a moderate distance from the shore in Jingjiang section.

**FIGURE 6:**
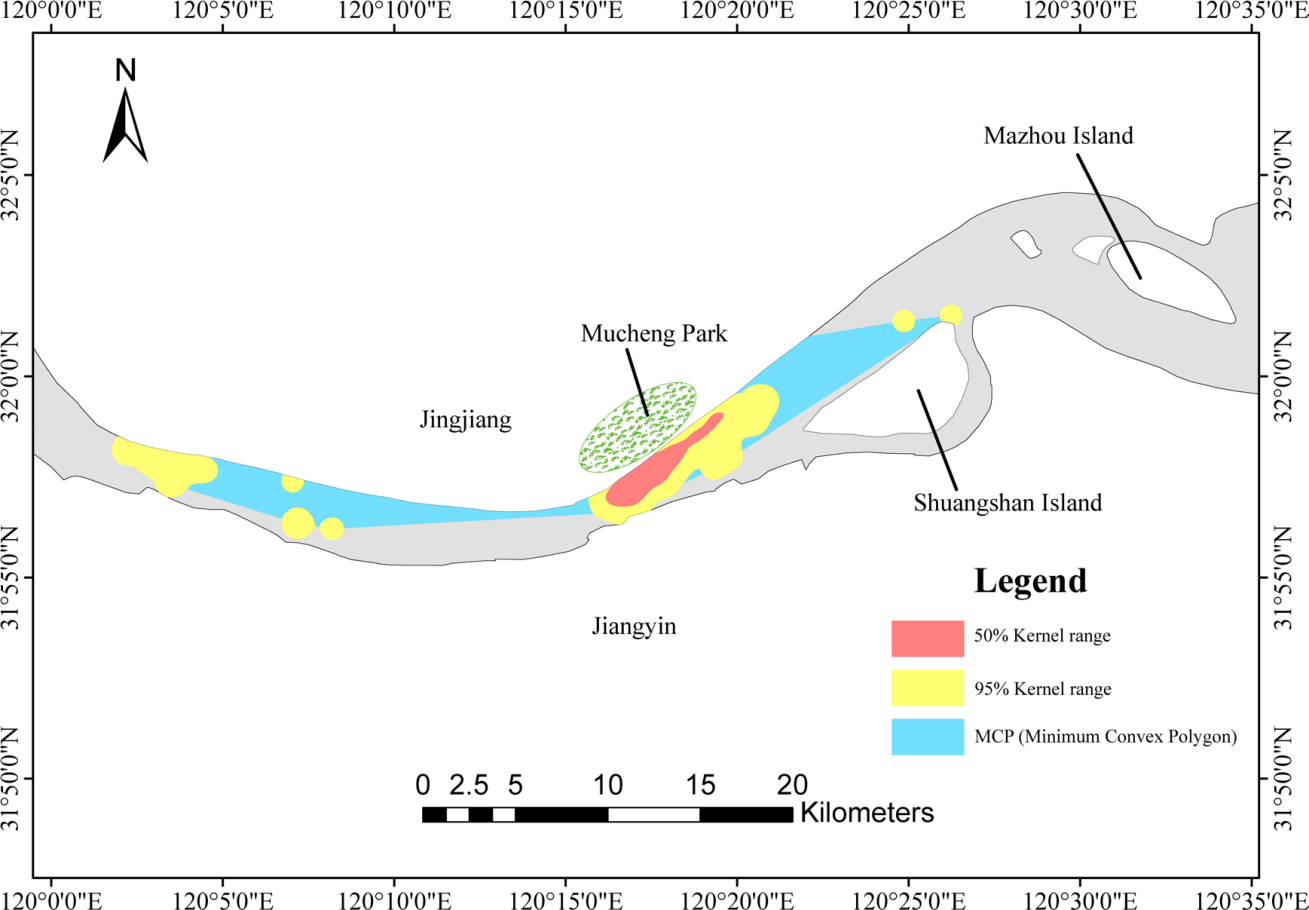
Habitat range of Yangtze finless porpoises in Jingjiang section.

The habitat range along the river section was extensive, covering areas from Jingjiang Yangtze River Bridge to Mazhou Island. The minimum convex polygon (MCP) range accounts for approximately 45% of the surveyed area. To be specific, the MCP range, 95% kernel density range, and 50% kernel density range were 83.63 km^2^, 43.93 km^2^, and 7.94 km^2^, respectively (Figure 6). However, the core habitats (50% KDE range) habitat range was primarily concentrated near Mucheng Park and Jingjiang Yangtze River Bridge.

### 3.4 Abundance Estimate

Based on the AIC values of the optimal models, the Hazard/Cosine, Hazard/Simple Polynomial (Hazard/SP), and Hazard/Hermite Polynomial (Hazard/HP) models all had the lowest AIC value of 1148.62. A plot of the detection function fitted to the frequency histogram of distance data showed a slowly decreasing trend as the distance from the trackline increases (Figure S1). The best model provided a good absolute fit, as assessed by the Q‐Q plot the weighted chi‐square goodness‐of‐fit tests (*p* = 0.563) (Figure S2). The density was 0.47 porpoises/km^2^(g(0) = 0.93, 95% CI = 0.35–0.62, % CV = 14.9), with an effective strip width (ESW) of 380.57 m (Table 2). If the MCP range was treated as the effective distribution range, about 39 (95% CI = 29–52, % CV = 14.9) individuals were distributed in the Jingjiang section. Furthermore, the population density of porpoises has an increasing trend over the years (in 2021–2022: 0.33 porpoises/km^2^, 95% CI = 0.19–0.58; in 2023: 0.44 porpoises/km^2^, 95% CI = 0.28–0.69; in 2024: 0.57 porpoises/km^2^, 95% CI = 0.37–0.89).

**TABLE 2:** Density estimation of Yangtze finless porpoises in Jingjiang section estimated by the line transect method.

Year	Model	NO. of para	AIC	Delta AIC	D (porpoise/km^2^)	95% CI	% CV	ESW (m)
2021–2024	HR/Cosine	2	1153.05	4.43	0.49	0.32–0.73	21.1	367.26
	HR/SP	1	1154.72	6.09	0.55	0.40–0.74	15.4	324.67
	HR/HP	2	1153.72	5.10	0.48	0.32–0.73	21.3	369.56
	Hazard/Cosine	2	1148.62	0	0.47	0.35–0.62	14.9	380.57
	Hazard/SP	2	1148.62	0	0.47	0.35–0.62	14.9	380.57
	Hazard/HP	2	1148.62	0	0.47	0.35–0.62	14.9	380.57
	NE/Cosine	2	1157.09	8.46	0.54	0.35–0.82	22.1	329.51
	NE/SP	3	1155.93	7.31	0.50	0.33–0.78	22.4	352.00
	NE/HP	2	1163.15	14.52	0.61	0.40–0.94	22.0	278.93
2021–2022	Hazard/Cosine	2	279.22	/	0.33	0.19–0.58	28.1	424.84
2023	Hazard/Cosine	2	301.43	/	0.44	0.28–0.69	22.5	334.08
2024	Hazard/Cosine	2	568.52	/	0.57	0.37–0.89	22.6	364.84

Abbreviations: CI, confidence interval; CV, coefficient of variation; D, density; ESW, effective strip width; HN, half‐rate; NE, negative polynomial; NO. of para, number of parameters; SP, simple polynomial.

## 4 Discussion

This study investigated the abundance and distribution of Yangtze finless porpoises in the Jingjiang section using questionnaire and boat survey. Both approaches yielded consistent conclusions regarding the distribution of porpoises, which were found primarily concentrated in the waters off Mucheng Park. However, the group sizes reported in questionnaire surveys were larger than those recorded during boat surveys. Additionally, both survey methods confirmed calf sightings in April, with calves also being detected during other months (February, March, and July) in the boat surveys.

### 4.1 Abundance

A zigzag or parallel transect design (with transect lines oriented at 45° to the channel centerline) in confined waters can provide better coverage of the study area (Dawson et al. 2008). However, the design is not feasible in the Yangtze River due to the higher vessel density (Zhao et al. 2008). Thus, transect lines parallel to the river bank were adopted. Such a sample line design has been successfully used several times in the estimation of porpoises in the Yangtze River (Zhao et al. 2008; Mei et al. 2014; Huang et al. 2020). In addition, the line transect sampling method is based on three fundamental assumptions, which are: (1) perfect detection on the trackline; (2) accurate distance measurement; (3) instantaneous detection at initial location (Buckland et al. 2001). If these conditions are not met, the estimation of abundance will exhibit significant bias (Buckland et al. 2001). For the first assumption, an independent observer performed data recording on the aft deck of the boat and was used for g(0) assessment in this study. A higher g(0) (0.93) was calculated in this survey than for previous surveys of Yangtze finless porpoise (range from 0.39 to 0.87) (Zhao et al. 2008; Mei et al. 2014; Huang et al. 2020; Lu et al. 2023). For small cetaceans surveyed by vessels, the g(0) values less than 1 are mostly due to perception bias (Dawson et al. 2008). Besides, the Yangtze finless porpoises have a respiratory interval of 15–20 s (Xiao and Wang 2006). During this time interval, the survey boat traveled 40–55 m with a speed of 10 km/h, ensuring that the porpoises would surface at least once before boat passage. Therefore, the visibility bias could be ignored for density estimation in this study. For the second assumption, all observers were trained prior to the survey, particularly in distance estimation. Moreover, geographical coordinates of boat and porpoise sightings were recorded during the surveys, and then the distance was verified using ArcMap 10.2. For the third assumption, simulation revealed that bias was negligible if mean animal speed was one quarter of that of the observer for whale surveys (Hiby 1986). The survey boat in this study traveled with a speed of 10 km/h, while the surface swimming speed of finless porpoises was measured at approximately 0.8 m/s (2.88 km/h) (Akamatsu et al. 2002). Therefore, the effect of porpoise movement on the results was negligible. However, the decreased detection rate of porpoises close to the transect line indicated a certain degree of vessel‐avoidance behavior. Bias in density estimation arises when animals exhibit avoidance behavior (resulting in underestimation) or attraction (leading to overestimation) relative to the transect line (Turnock and Quinn 1991; Palka and Hammond 2001). Consequently, the density estimates of porpoises in this study may be underestimated.

The middle and lower regions of the Yangtze River used to be the main distribution area of the Yangtze finless porpoise, especially from Ezhou to Jiangyin (Zhang et al. 1993; Zhao et al. 2008). However, the population experienced a sharp decline in abundance, and its distribution became fragmented just a few years later (Mei et al. 2014). The 2017 survey further confirmed the existence of multiple low‐density zones, including the Jingjiang section (Huang et al. 2020). However, our survey showed that there are about 39 porpoises living in the Jingjiang section, which is higher than previous reports. This may be due to lower survey effort in previous surveys of porpoises in the Yangtze River, which was 38 days in 2006, 44 days in 2012, and 37 days in 2017 (these surveys covered the section from Yichang to Shanghai and only several days in the Jingjiang) (Zhao et al. 2008; Mei et al. 2014; Huang et al. 2020). In the present study, a 115‐day survey was carried out and was the largest survey effort in the region from 2021 to 2024, and this is also the first time that a special survey has been conducted in this section. Additionally, porpoises showed an increase in density from 2021 to 2024, which could be related to the Yangtze River conservation and the fishing ban policy. In summary, the population abundance of Yangtze finless porpoise may have been seriously underestimated in the Jingjiang section for a long time.

### 4.2 Distribution

The distribution of the finless porpoises in Jingjiang is concentrated at an offshore distance of 200–800 m, with less than 10% observed within 300 m. This significantly differs from previous studies indicating that finless porpoises primarily inhabit areas within 300 m from the shore (probably more than 80%) (Zhang et al. 1993; Zhou et al. 1998; Wei et al. 2003). The difference in distribution may be related to variations in river width across different sections of the Yangtze River. The river width from Yichang to Wuhan is 1500–2000 m, from Wuhan to Jiangyin is 2000–3000 m, and from Jiangyin to Wusongkou is over 3500 m (Zhang et al. 1993). The widest section of the Jingjiang can reach up to 6000 m. The second reason may be that previous studies had fewer sightings in the Jingjiang, probably missing the porpoises further.

The Jingjiang population prefers natural river banks or areas with less human interference, such as the Mucheng Park waters. This is similar to previous findings that the Yangtze finless porpoises exhibit a strong preference for sandbanks, slow current waters, natural river banks, and confluence areas (Dong et al. 2015; Mei et al. 2017; Chen et al. 2020; Lu et al. 2023). Other cetaceans living in river systems have the same characteristics, such as the Amazon dolphin, *Inia geoffrensis* (Gomez‐Salazar et al. 2012) and the Irrawaddy dolphin, *Orcaella brevirostris* (Braulik et al. 2012).

### 4.3 Conservation Enlightenments

The abundance of Yangtze finless porpoises has been reported several times and has generally been showing a decreasing trend (Zhang et al. 1993; Zhao et al. 2008; Mei et al. 2014; Huang et al. 2020). Despite that the conservation measures already taken has halted the negative trend and a slight increase in population numbers, the current protection measures are still inadequate. Zhao et al. (2013) revealed that existing protected areas do not fully encompass the high‐density range of porpoises, despite the establishment of the Nanjing reserve in 2014 (30.9% of the finless porpoise's habitat is protected). Huang et al. (2017) claimed that the Yangtze finless porpoises urgently needed in situ protection measures to prevent their extinction. The current study shows that the abundance of porpoises in the Jingjiang section significantly exceed previous estimates, indicating that in situ conservation of this area should be prioritized. Additionally, surveys of other low‐density areas should be expanded to prevent underestimating abundance due to insufficient survey efforts. Consistent with the findings of previous studies, the current research further confirms that the Yangtze finless porpoise prefers environments with minimal anthropogenic disturbance.

## 5 Conclusion

The declining trend of Yangtze finless porpoises has been alleviated, and their population abundance has increased (Huang et al. 2020; Hao et al. 2023). An estimated 39 porpoises have been recorded in the Jingjiang section, significantly surpassing previous reports (Huang et al. 2020). These results suggest that conservation efforts for the Yangtze finless porpoise have had a positive impact. Therefore, additional survey efforts are needed in certain sections of the Yangtze River, and further habitat protection measures should be implemented.

## Author Contributions

**Yi Lu:** conceptualization (equal), data curation (lead), investigation (equal), methodology (lead), software (lead), writing – original draft (lead), writing – review and editing (equal). **Hongyan Bi:** data curation (equal), investigation (supporting), methodology (supporting), writing – original draft (supporting), writing – review and editing (supporting). **Weijie Jiang:** data curation (supporting), investigation (supporting), methodology (supporting), software (supporting), writing – original draft (supporting), writing – review and editing (supporting). **Chong Cui:** data curation (supporting), methodology (supporting), software (supporting). **Jing Zhu:** data curation (supporting), methodology (supporting), software (supporting). **Pei Wang:** investigation (supporting), methodology (supporting), writing – original draft (supporting). **Xinrong Xu:** data curation (equal), methodology (supporting). **Guang Yang:** formal analysis (supporting), project administration (equal), supervision (equal), writing – review and editing (supporting). **Wei Chen:** conceptualization (equal), funding acquisition (lead), project administration (equal). **Zhengfei Wang:** conceptualization (equal), investigation (equal), methodology (supporting), project administration (equal), supervision (equal), writing – review and editing (supporting).

## Conflicts of Interest

The authors declare no conflicts of interest.

## Supporting information


**Figure S1:** Histogram of the distance data and the corresponding best model (Hazard/Cosine) among the set of candidate models.

**Figure S2:** Quantile‐quantile (Q‐Q) plot corresponding to fit of the Hazard + Cosine model to line transect data.

## Data Availability

All the required data are uploaded as Supporting Information—S1.

## References

[ece371649-bib-0001] Akamatsu, T. , D. Wang , K. Wang , Z. Wei , Q. Zhao , and Y. Naito . 2002. “Diving Behaviour of Freshwater Finless Porpoises (*Neophocaena phocaenoides*) in an Oxbow of the Yangtze River, China.” ICES Journal of Marine Science 59, no. 2: 438–443. 10.1006/jmsc.2001.1159.

[ece371649-bib-0002] Barlow, J. 1995. “The Abundance of Cetaceans in California Waters. Part I: Ship Surveys in Summer and Fall of 1991.” Fishery Bulletin 93: 1–14. https://spo.nmfs.noaa.gov/sites/default/fles/pdf‐content/1995/931/barlow.pdf.

[ece371649-bib-0003] Barlow, J. , and K. A. Forney . 2007. “Abundance and Population Density of Cetaceans in the California Current Ecosystem.” Fishery Bulletin 105: 509–526. http://fshbull.noaa.gov/1054/barlow.pdf.

[ece371649-bib-0005] Braulik, G. T. , A. P. Reichert , T. Ehsan , et al. 2012. “Habitat Use by a Freshwater Dolphin in the Low‐Water Season.” Aquatic Conservation: Marine and Freshwater Ecosystems 22, no. 4: 533–546. 10.1002/aqc.2246.

[ece371649-bib-0006] Buckland, S. T. , D. R. Anderson , K. P. Burnham , J. L. Laake , D. L. Borchers , and L. Thomas . 2001. Introduction to Distance Sampling. Oxford University Press.

[ece371649-bib-0007] Burnham, K. P. , S. T. Buckland , J. L. Laake , et al. 2004. “Further Topics in Distance Sampling.” In Advanced Distance Sampling: Estimating Abundance of Biological Populations, edited by S. T. Buckland , D. R. Anderson , K. P. Burnham , J. L. Laake , D. L. Borchers , and L. Thomas , 307–392. Oxford University Press.

[ece371649-bib-0010] Chen, M. , D. Yu , Y. Lian , and Z. Liu . 2020. “Population Abundance and Habitat Preference of the Yangtze Finless Porpoise in the Highest Density Section of the Yangtze River.” Aquatic Conservation: Marine and Freshwater Ecosystems 30, no. 6: 1088–1097. 10.1002/aqc.3299.

[ece371649-bib-0011] Dawson, S. , P. Wade , E. Slooten , and J. A. Y. Barlow . 2008. “Design and Field Methods for Sighting Surveys of Cetaceans in Coastal and Riverine Habitats.” Mammal Review 38, no. 1: 19–49. https://digitalcommons.unl.edu/usdeptcommercepub/255.

[ece371649-bib-0012] Dong, L. , D. Wang , K. Wang , et al. 2015. “Yangtze Finless Porpoises Along the Main Channel of Poyang Lake, China: Implications for Conservation.” Marine Mammal Science 31, no. 2: 612–628. 10.1111/mms.12181.

[ece371649-bib-0013] Evans, P. G. H. , and P. S. Hammond . 2010. “Monitoring Cetaceans in European Waters.” Mammal Review 34, no. 1–2: 131–156. 10.1046/j.0305-1838.2003.00027.x.

[ece371649-bib-0015] Focardi, S. , R. Isotti , and A. Tinelli . 2002. “Line Transect Estimates of Ungulate Populations in a Mediterranean Forest.” Journal of Wildlife Management 66, no. 1: 48–58. 10.2307/3802870.

[ece371649-bib-0017] Gomez‐Salazar, C. , F. Trujillo , M. Portocarrero‐Aya , and H. Whitehead . 2012. “Population, Density Estimates, and Conservation of River Dolphins (Inia and Sotalia) in the Amazon and Orinoco River Basins.” Marine Mammal Science 28, no. 1: 124–153. 10.1111/j.1748-7692.2011.00468.x.

[ece371649-bib-0019] Hao, Y. , K. Wang , G. Nabi , J. S. Zheng , K. X. Wang , and D. Wang . 2023. “Recent Progress and Future Directions for Conservation of the Yangtze Finless Porpoise (Neophocaena Asiaorientalis Asiaorientalis).” Der Zoologische Garten 91: 155–173. 10.53188/zg0021.

[ece371649-bib-0020] Hao, Y. J. , B. Tang , Z. G. Mei , J. S. Zheng , K. X. Wang , and D. Wang . 2024. “A Retrospective Analysis of the Conservation Progress of the Yangtze Finless Porpoise and Suggestions for Further Conservation.” Journal of Hydrobiology 48, no. 6: 1065–1072. https://link.cnki.net/urlid/42.1230.Q20240319.0910.002.

[ece371649-bib-0021] Hiby, A. R. 1986. “Results of a Hazard Rate Model Relevant to Experiments on the 1984/85 IDCR Minke Whale Assessment Cruise.” Report of the International Whaling Commission 36: 497–498.

[ece371649-bib-0022] Huang, J. , Z. Mei , M. Chen , et al. 2020. “Population Survey Showing Hope for Population Recovery of the Critically Endangered Yangtze Finless Porpoise.” Biological Conservation 241: 108315. 10.1016/j.biocon.2019.108315.

[ece371649-bib-0023] Huang, S. L. , Z. Mei , Y. Hao , J. Zheng , K. Wang , and D. Wang . 2017. “Saving the Yangtze Finless Porpoise: Time Is Rapidly Running out.” Biological Conservation 210: 40–46. 10.1016/j.biocon.2016.05.021.

[ece371649-bib-0024] Li, D. , D. Q. Lin , Z. G. Wang , et al. 2024. “Exploring the Relationship Between the Population Distribution of Yangtze Finless Porpoise and Underwater Noise in Zhenjiang Yangtze River Porpoise Provincial Nature Reserve, Jiangsu Province.” Journal of Hydrobiology 48, no. 10: 1660–1671. https://creativecommons.org/licenses/by/4.0.

[ece371649-bib-0025] Liu, M. , L. Bejder , M. Lin , P. Zhang , L. Dong , and S. Li . 2020. “Determining Spatial Use of the World's Second Largest Humpback Dolphin Population: Implications for Place‐Based Conservation and Management.” Aquatic Conservation: Marine and Freshwater Ecosystems 30, no. 2: 364–374. 10.1002/aqc.3253.

[ece371649-bib-0026] Lu, Y. , X. Xu , B. Chen , and G. Yang . 2023. “The Identification of Critically Endangered Yangtze Finless Porpoise High‐Density Areas for Priority Protection.” Mammalian Biology 103: 277–287. 10.1007/S42991-023-00350-X.

[ece371649-bib-0028] Mei, Z. G. , M. Chen , Y. T. Li , et al. 2017. “Habitat Preference of the Yangtze Finless Porpoise in a Minimally Disturbed Environment.” Ecological Modelling 353: 47–53. 10.1016/j.ecolmodel.2016.12.020.

[ece371649-bib-0029] Mei, Z. G. , Y. J. Hao , J. S. Zhang , Z. T. Wang , K. X. Wang , and D. Wang . 2021. “Population Status and Conservation Outlooks of Yangtze Finless Porpoise in the Lake Poyang.” Journal of Lake Science 33, no. 5: 1289–1298.

[ece371649-bib-0030] Mei, Z. G. , Y. J. Hao , J. S. Zheng , K. X. Wang , S. H. Li , and N. Wang . 2011. “Research Progress on the Population Decline Mechanism of the Yangtze Finless Porpoise.” Life Sciences 5: 519–524. 10.13376/j.cbls/2011.05.018.

[ece371649-bib-0032] Mei, Z. G. , X. Q. Zhang , S. L. Huang , et al. 2014. “The Yangtze Finless Porpoise: On an Accelerating Path to Extinction?” Biological Conservation 172: 117–123. 10.1016/j.biocon.2014.02.033.

[ece371649-bib-0035] Palka, D. L. , and P. S. Hammond . 2001. “Accounting for Responsive Movement in Line Transect Estimates of Abundance.” Canadian Journal of Fisheries and Aquatic Sciences 58, no. 4: 777–787. 10.1139/cjfas-58-4-777.

[ece371649-bib-0036] Shan, Y. 2019. “15 Nature Reserves Cover 40% of the Distribution Area of the Yangtze River Dolphin–The 10th International Freshwater Dolphin Day Celebration Was Held in Yangzhou.” China Fisheries 11: 24.

[ece371649-bib-0037] Song, X. Z. , T. Xia , and C. X. Xu . 2017. “The World's First Reintroduction of a Captive‐Bred Yangtze Finless Porpoise Into the Wild.” Fisheries Science & Technology Information 44, no. 3: 164.

[ece371649-bib-0038] Sprogis, K. R. , H. C. Raudino , R. Rankin , C. D. MacLeod , and L. Bejder . 2016. “Home Range Size of Adult Indo‐Pacific Bottlenose Dolphins ( *Tursiops aduncus* ) in a Coastal and Estuarine System Is Habitat and Sex‐Specific.” Marine Mammal Science 32, no. 1: 287–308. 10.1111/mms.12260.

[ece371649-bib-0039] Stober, J. M. , and L. L. Smith . 2010. “Total Counts Versus Line Transects for Estimating Abundance of Small Gopher Tortoise Populations.” Journal of Wildlife Management 74, no. 7: 1595–1600. 10.1111/j.1937-2817.2010.tb01289.x.

[ece371649-bib-0040] Turnock, B. J. , and T. J. Quinn . 1991. “The Effect of Responsive Movement on Abundance Estimation Using Line Transect Sampling.” Biometrics 47, no. 2: 701–715. http://www.jstor.org/stable/2532156.

[ece371649-bib-0041] Turvey, S. T. , R. L. Pitman , B. L. Taylor , et al. 2007. “First Human‐Caused Extinction of a Cetacean Species?” Biology Letters 3, no. 5: 537–540. 10.1098/rsbl.2007.0292. 17686754 PMC2391192

[ece371649-bib-0043] Wang, D. , Y. Hao , K. Wang , et al. 2005. “Aquatic Resource Conservation: The First Yangtze Finless Porpoise Successfully Born in Captivity.” Environmental Science and Pollution Research International 12: 247–250. 10.1065/espr2005.08.284. 16206715

[ece371649-bib-0046] Wang, R. , Y. Han , F. Fan , et al. 2022. “Need to Shift in River‐Lake Connection Scheme Under the “Ten‐Year Fishing Ban” in the Yangtze River, China.” Ecological Indicators 143: 109434. 10.1016/j.ecolind.2022.109434.

[ece371649-bib-0047] Wang, X. , F. Wu , Q. Zhu , and S. L. Huang . 2017. “Long‐Term Changes in the Distribution and Core Habitat Use of a Coastal Delphinid in Response to Anthropogenic Coastal Alterations.” Aquatic Conservation: Marine and Freshwater Ecosystems 27, no. 3: 643–652. 10.1002/aqc.2720.

[ece371649-bib-0048] Wei, Z. , X. F. Zhang , K. X. Wang , et al. 2003. “Habitat Use and Preliminary Evaluation of the Habitat Status of the Yangtze Finless Porpoise (*Neophocaena Phocaenoides Asiaeorientalis*) in the Balijiang Section of the Yangtze River.” Acta Zoologica Sinica 49, no. 2: 163–170.

[ece371649-bib-0049] Wilson, G. J. , and R. J. Delahay . 2001. “A Review of Methods to Estimate the Abundance of Terrestrial Carnivores Using Field Signs and Observation.” Wildlife Research 28, no. 2: 151–164. 10.1071/WR00033.

[ece371649-bib-0050] Xiao, J. , and D. Wang . 2006. “Respiratory Pattern of Captive Yangtze Finless Porpoises ( *Neophocaena phocaenoides asiaeorientalis* ).” Journal of Ethology 24: 205–212. 10.1007/s10164-005-0181-3.

[ece371649-bib-0051] Xiao, W. , and X. F. Zhang . 2000. “A Preliminary Study on the Population Size of Yangtze Finless Porpoise in Poyang Lake.” Biodiversity Science 2000, no. 1: 106–111.

[ece371649-bib-0052] Zhang, X. , D. Wang , R. Liu , et al. 2003. “The Yangtze River Dolphin or Baiji (*Lipotes vexillifer*): Population Status and Conserva Tion Issues in the Yangtze River, China.” Aquatic Conservation: Marine and Freshwater Ecosystems 13, no. 1: 5–64. 10.1002/aqc.547.

[ece371649-bib-0053] Zhang, X. F. , R. J. Liu , Q. Z. Zhao , et al. 1993. “Evaluation of the Current Status of Finless Porpoise Populations in the Middle and Lower Reaches of the Yangtze River.” Acta Theriologica Sinica 4: 260–270. 10.16829/j.slxb.1993.04.005.

[ece371649-bib-0054] Zhao, X. , J. Barlow , B. L. Taylor , et al. 2008. “Abundance and Conservation Status of the Yangtze Finless Porpoise in the Yangtze River China.” Biological Conservation 141, no. 12: 3006–3018. 10.1016/j.biocon.2008.09.005.

[ece371649-bib-0055] Zhao, X. , D. Wang , S. T. Turvey , B. Taylor , and T. Akamatsu . 2013. “Distribution Patterns of Yangtze Finless Porpoises in the Yangtze River: Implications for Reserve Management.” Animal Conservation 16, no. 5: 509–518. 10.1111/acv.12019.

[ece371649-bib-0057] Zhou, K. , G. Yang , A. Gao , J. Sun , and X. Xu . 1998. “Population Abundance and Distribution Characteristics of Finless Porpoise in the River Section From Nanjing to Hukou of the Yangtze River.” Journal of Nanjing Normal University 21: 91–98.

[ece371649-bib-0058] Zhou, Y. , Y. Chen , and X. Li . 2016. “Application of the Sample Vessel Baseline Method in the Yangtze River Fishery Resource Survey.” Journal of Fishery Sciences of China 23, no. 3: 451–459.

