# Supplementary figures and images for: Two Strategies for Researching the Endangered Yangtze Finless Porpoises Suggest Data‐Poor Areas Are Worthy of Greater Conservation Efforts
Source: Ecol Evol. 2025 Jun 26;15(7):e71649. doi: 10.1002/ece3.71649 (PMC12202776; doi:10.1002/ece3.71649)

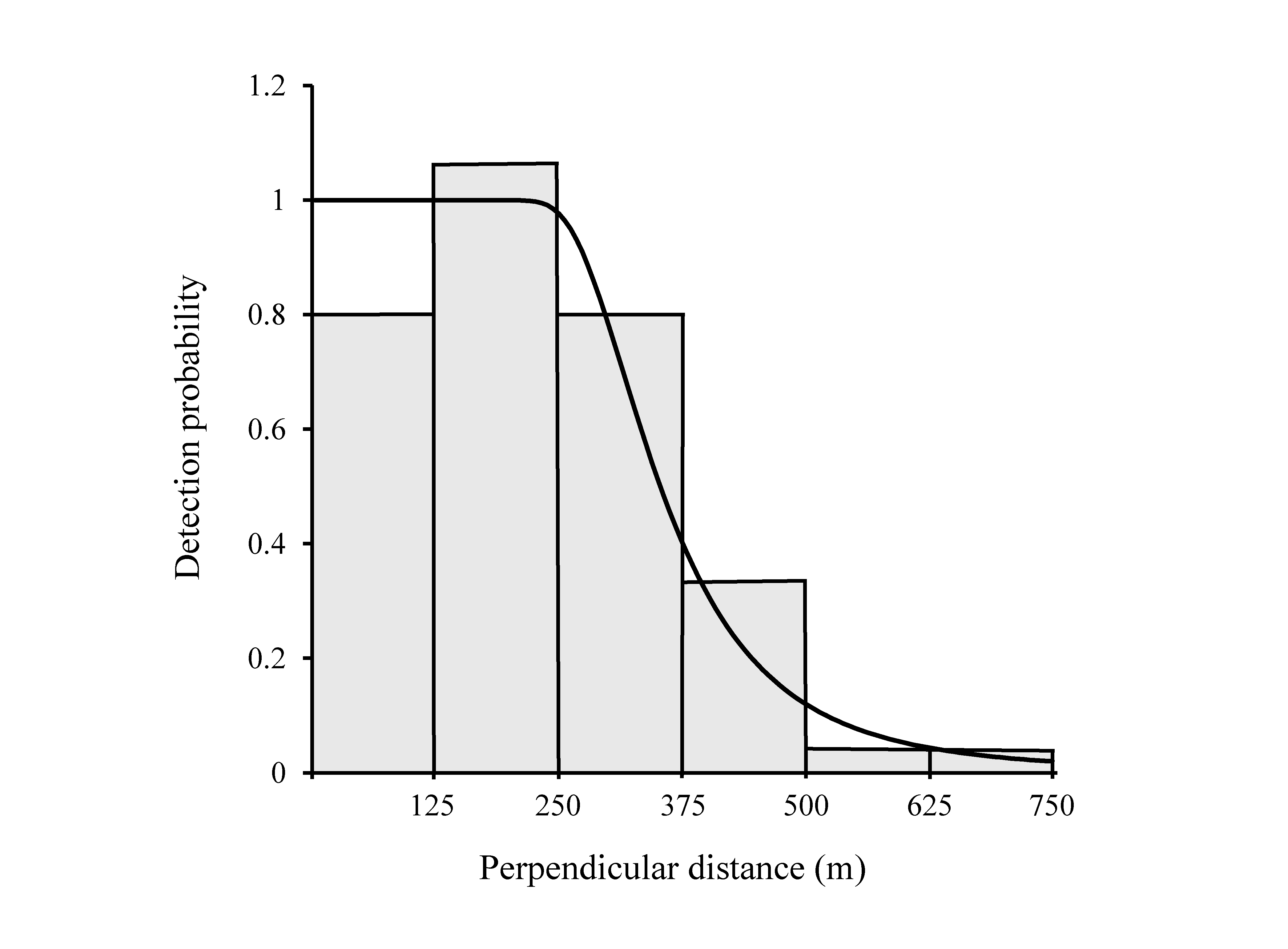

Supplement: Supplementary file 1 — Figure S1: Histogram of the distance data and the corresponding best model (Hazard/Cosine) among the set of candidate models. [file ECE3-15-e71649-s001.jpg]

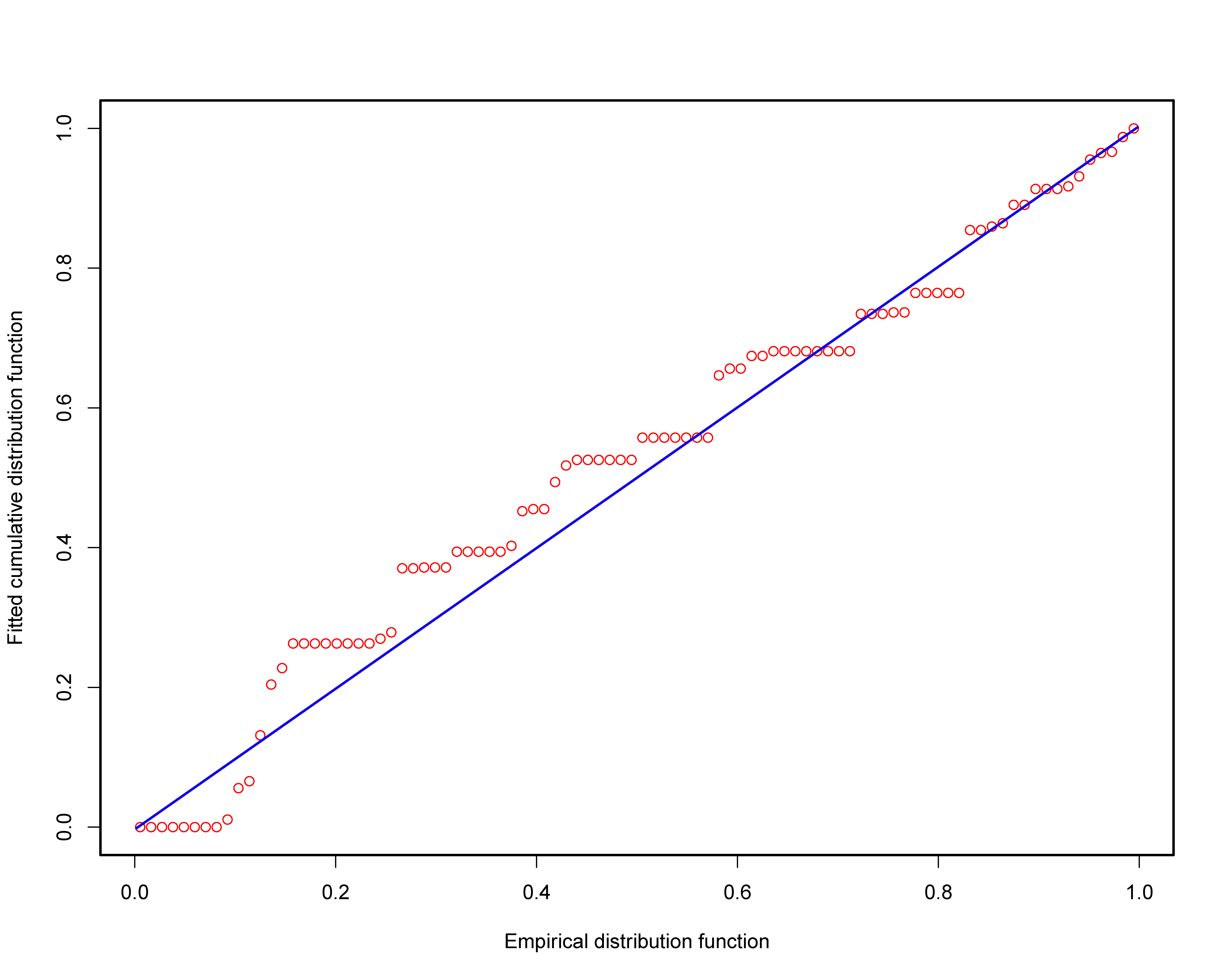

Supplement: Supplementary file 2 — Figure S2: Quantile‐quantile (Q‐Q) plot corresponding to fit of the Hazard + Cosine model to line transect data. [file ECE3-15-e71649-s002.jpg]
